# Supplementary material for: Cost-Effective Cultivation of Native PGPB Sinorhizobium Strains in a Homemade Bioreactor for Enhanced Plant Growth
Source: Bioengineering (Basel). 2023 Aug 13;10(8):960. doi: 10.3390/bioengineering10080960 (PMC10451550; doi:10.3390/bioengineering10080960)
Supplement: Supplementary file 1 [file bioengineering-10-00960-s001.zip › Figure S1-Supplementary material.docx]

**Supplementary material**

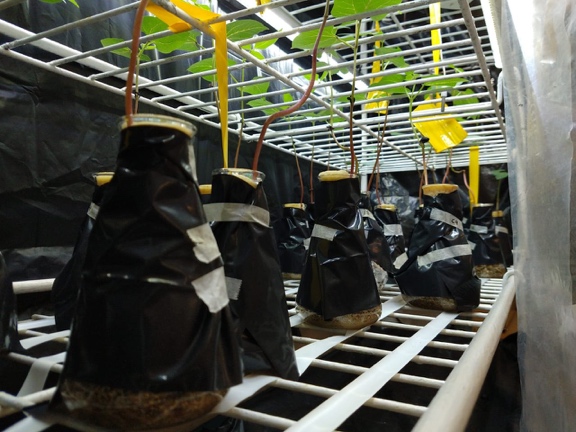

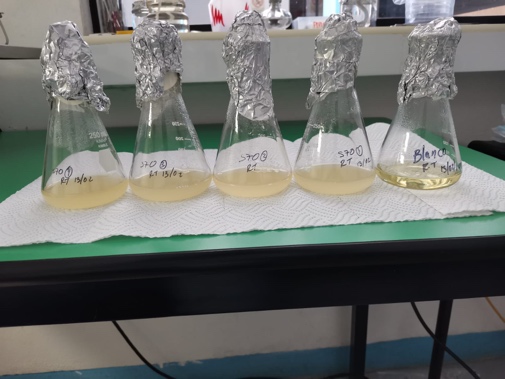


**Bacterial inoculum (Treatments)**

**Test plants (*Phaseolus vulgaris*)**

**Figure S1**. Inoculation test on common bean plants (*Phaseolus vulgaris*) treated with *Sinorhizobium* bacteria (inoculation treatments).
