# Supplementary figures and images for: Cost-Effective Cultivation of Native PGPB Sinorhizobium Strains in a Homemade Bioreactor for Enhanced Plant Growth
Source: Bioengineering (Basel). 2023 Aug 13;10(8):960. doi: 10.3390/bioengineering10080960 (PMC10451550; doi:10.3390/bioengineering10080960)

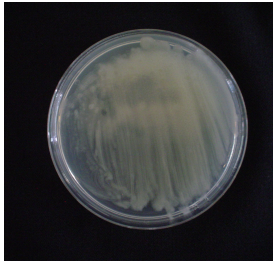

*Sinorhizobium* strain

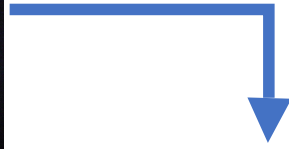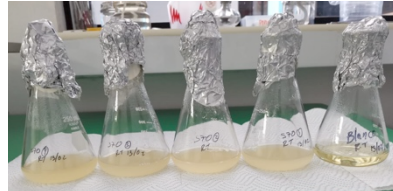

**Bacterial inoculum  
(Treatments)**

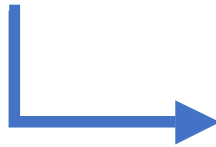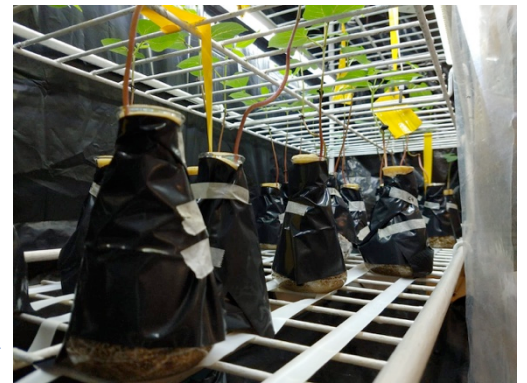

**Test plants  
(*Phaseolus vulgaris*)**

Supplement: Supplementary file 1 [file bioengineering-10-00960-s001.zip › Figure S1.pdf]
